# Thermally Tunable Ultra-Broadband Terahertz Metasurface Absorber Based on VO_2_ Phase Transition

**DOI:** 10.3390/nano16201293

**Published:** 2026-10-12

**Authors:** Ahmed Ali, Hamid Sharif, Asrafali Barkathulla, Wazie M. Abdulkawi, Majeed A. S. Alkanhal, Yosef T. Aladadi

**Affiliations:** 1 College of Physics and Optoelectronic Engineering, Shenzhen University, Shenzhen 518060, China; ahmedali23@email.szu.edu.cn; 2 Shenzhen Key Laboratory of Micro-Nano Photonic Information Technology, Key Laboratory of Optoelectronic Devices and Systems of Ministry of Education and Guangdong Province, College of Physics and Optoelectronic Engineering, THz Technical Research Center of Shenzhen University, Shenzhen University, Shenzhen 518060, China; 3 Department of Electrical Engineering, College of Engineering in Wadi Addawasir, Prince Sattam Bin Abdulaziz University, Al-Kharj 11991, Saudi Arabia; 4 Department of Electrical Engineering, College of Engineering, King Saud University, Riyadh 11421, Saudi Arabia; 5 Department of Electrical Engineering, College of Engineering, Imam Mohammad Ibn Saud Islamic University (IMSIU), Riyadh 11432, Saudi Arabia

**Keywords:** terahertz, metamaterial absorber, tunable, broadband, vanadium dioxide

## Abstract

Vanadium dioxide (VO_2_), owing to its reversible insulator-to-metal phase transition, is a promising material for tunable terahertz (THz) devices. In this work, a compact VO_2_-based terahertz absorber is numerically proposed to achieve thermally tunable ultra-broadband absorption. The proposed single-layer metasurface employs a fourfold-symmetric integration of cross-shaped, circular-ring, and square-frame elements to induce multiple coupled resonances and their spectral overlap, providing a design strategy for broadening the absorption bandwidth while maintaining polarization-insensitive operation. The optimized structure exhibits five near-perfect absorption resonances at 3.23, 5.27, 7.34, 8.82, and 9.73 THz. At a VO_2_ conductivity of 2.0 × 10^5^ S/m, the absorber achieves absorptance exceeding 90% from 2.95 to 10.36 THz, corresponding to a bandwidth of 7.41 THz and a relative bandwidth of 111.35%. The absorption mechanism is attributed to the hybridization of multiple resonant modes and effective impedance matching with free space, as confirmed by electromagnetic field and impedance analyses. Furthermore, the absorption response can be dynamically tuned through the phase transition of VO_2_. The absorber also exhibits polarization-insensitive behavior and maintains high absorptance over a wide range of incident angles. These results demonstrate a compact and thermally reconfigurable platform for terahertz imaging, sensing, spectroscopy, and adaptive electromagnetic stealth.

## 1. Introduction

The terahertz (THz) frequency spectrum, generally spanning 0.1–10 THz, occupies the transitional region between the microwave and infrared bands and has emerged as a key area of research for next-generation electromagnetic technologies [1,2,3,4]. The increasing interest in THz technology is primarily attributed to its unique electromagnetic characteristics, including ultra-wide spectral bandwidth, low photon energy, non-ionizing interaction with biological tissues, and high sensitivity to the physical and chemical properties of materials. These distinctive features make THz waves highly attractive for a broad range of applications, including ultra-high-speed wireless communications [5], security screening and concealed-object detection [6], and biomedical imaging and diagnostics [7], as well as non-destructive testing and material characterization [8]. To fully exploit these advantages, substantial research efforts have been devoted to the development of advanced THz devices based on artificially engineered metamaterials and metasurfaces. Among these devices, filters [9], modulators [10,11], and absorbers [12,13] have received considerable attention owing to their remarkable ability to manipulate electromagnetic waves with high flexibility and efficiency.

Among the various metamaterial-based devices, metamaterial absorbers have emerged as one of the most extensively investigated structures because of their ability to efficiently dissipate incident electromagnetic energy while simultaneously suppressing reflection and transmission. Through careful engineering of the effective electric and magnetic responses of subwavelength resonant structures, near-perfect impedance matching with free space can be achieved, leading to absorption levels approaching unity. Since the pioneering demonstration of the first perfect metamaterial absorber by Landy et al. in 2008 [14], extensive research has been conducted across microwave, terahertz, infrared, and optical frequency regimes. As a result, numerous absorber configurations have been reported, including single-band [15], dual-band [16], multi-band [17], and broadband designs [18,19,20,21,22]. Among these categories, broadband absorbers have attracted particular interest because of their ability to maintain high absorption performance over a wide frequency range. Such characteristics are highly desirable for various practical applications, including terahertz imaging [23], highly sensitive sensing systems [24], electromagnetic interference suppression, stealth technologies, and advanced electromagnetic wave control applications [25,26].

Despite the remarkable progress achieved in THz metamaterial absorbers, the realization of broadband absorption with consistently high efficiency remains a significant challenge. Most reported THz absorbers operate based on resonant electromagnetic mechanisms, where strong electromagnetic confinement occurs only within a limited frequency range, inherently restricting the achievable absorption bandwidth [27,28,29,30]. To overcome this limitation, numerous design strategies have been proposed to generate multiple resonances and promote their spectral overlap. Among the most widely adopted approaches are multilayer architectures and geometrically sophisticated unit-cell configurations capable of supporting multiple resonant modes simultaneously, thereby extending the effective absorption bandwidth [31,32,33]. Representative examples include multi-square metallic resonators [34], multi-strip patterned structures [35], and stacked concentric resonator configurations [31], all of which have demonstrated enhanced broadband absorption through resonance hybridization and coupling effects. Nevertheless, these approaches often require intricate geometrical features, increased structural thickness, or multiple fabrication steps, resulting in higher manufacturing complexity, increased cost, and greater sensitivity to fabrication tolerances. Consequently, the development of compact and structurally simple broadband absorbers capable of maintaining excellent absorption performance remains an important research objective in the terahertz regime.

In recent years, the incorporation of functional materials into metamaterial absorbers has emerged as an effective strategy for enhancing absorption performance while simultaneously enabling active reconfigurability [23,36,37,38]. Among the various materials explored for THz applications, graphene and vanadium dioxide (VO_2_) have attracted significant attention owing to their exceptional electromagnetic properties. Graphene enables dynamically controlled electromagnetic responses through modulation of its surface conductivity, making it a promising candidate for reconfigurable THz devices [26,39,40]. Meanwhile, VO_2_ has emerged as one of the most attractive phase-change materials because of its reversible insulator-to-metal transition near 68 °C, accompanied by a conductivity variation spanning several orders of magnitude [41,42]. This dramatic conductivity change enables efficient manipulation of electromagnetic waves and provides a versatile mechanism for realizing dynamically tunable absorption characteristics.

Besides VO_2_, chalcogenide phase-change materials based on germanium and antimony, such as Ge_2_Sb_2_Te_5_ (GST), Sb_2_Se_3_ and Sb_2_S_3_, have been widely studied for reconfigurable photonic devices owing to their nonvolatile switching between amorphous and crystalline states and their large refractive-index contrast [43,44]. However, their tunability mainly arises from refractive-index modulation at optical and near-infrared wavelengths, and their state transitions require high-temperature pulsed heating, together with careful thermal management and endurance considerations [43]. GST additionally exhibits relatively high optical loss, whereas the wide-bandgap antimony-based materials are mainly advantageous for low-loss phase modulation [43,44]. In contrast, VO_2_ undergoes a reversible insulator-to-metal transition at a modest temperature of about 68 °C, accompanied by a conductivity change of several orders of magnitude, which makes it particularly suitable for tuning the loss of terahertz absorbers. Its main limitation is volatility, as the metallic phase must be maintained by a continuous thermal, electrical or optical stimulus.

Leveraging these properties of VO_2_, numerous VO_2_-based metamaterial absorbers have been proposed in recent years. Several studies have demonstrated broadband absorption with absorptivity exceeding 90% across selected terahertz frequency ranges [45,46], while multifunctional metasurfaces capable of switching between distinct electromagnetic functionalities, such as absorption and polarization conversion, have also been reported [47]. Despite these encouraging developments, many existing VO_2_-based absorbers remain constrained by limited operating bandwidths, relatively low operating frequencies, complicated multilayer configurations, or intricate resonator geometries. Consequently, achieving ultra-broadband absorption while simultaneously preserving structural simplicity, compactness, and tunability remains a significant challenge for current VO_2_-based absorber technologies.

Motivated by these challenges, this work presents a compact VO_2_-based terahertz metamaterial absorber that combines ultra-broadband absorption, high efficiency, and thermally reconfigurable operation within a simple resonator architecture. The proposed structure consists of a patterned VO_2_ resonator comprising a square frame, concentric circular ring, and central cross element, separated from a metallic ground plane by a low-permittivity photonic crystal slab (PCS) spacer. By exploiting the thermally induced insulator-to-metal transition of VO_2_ and the hybridization of multiple resonant modes, the proposed absorber achieves five near-perfect absorption peaks located at 3.23, 5.27, 7.34, 8.82, and 9.73 THz. At a VO_2_ conductivity of 2 × 10^5^ S/m, the absorber exhibits absorption exceeding 90% over an ultra-wide frequency range from 2.95 to 10.36 THz, corresponding to a bandwidth of 7.41 THz and a relative bandwidth of 111.35%. Furthermore, the proposed structure maintains polarization-insensitive characteristics under normal incidence and stable absorption performance over a broad range of incident angles. The underlying absorption mechanism is systematically investigated through impedance matching analysis and near-field electromagnetic distributions. These results demonstrate a compact and thermally reconfigurable platform with promising potential for terahertz sensing, imaging, spectroscopy, and adaptive electromagnetic wave control.

### Design and Structural Configuration

**Figure 1:**
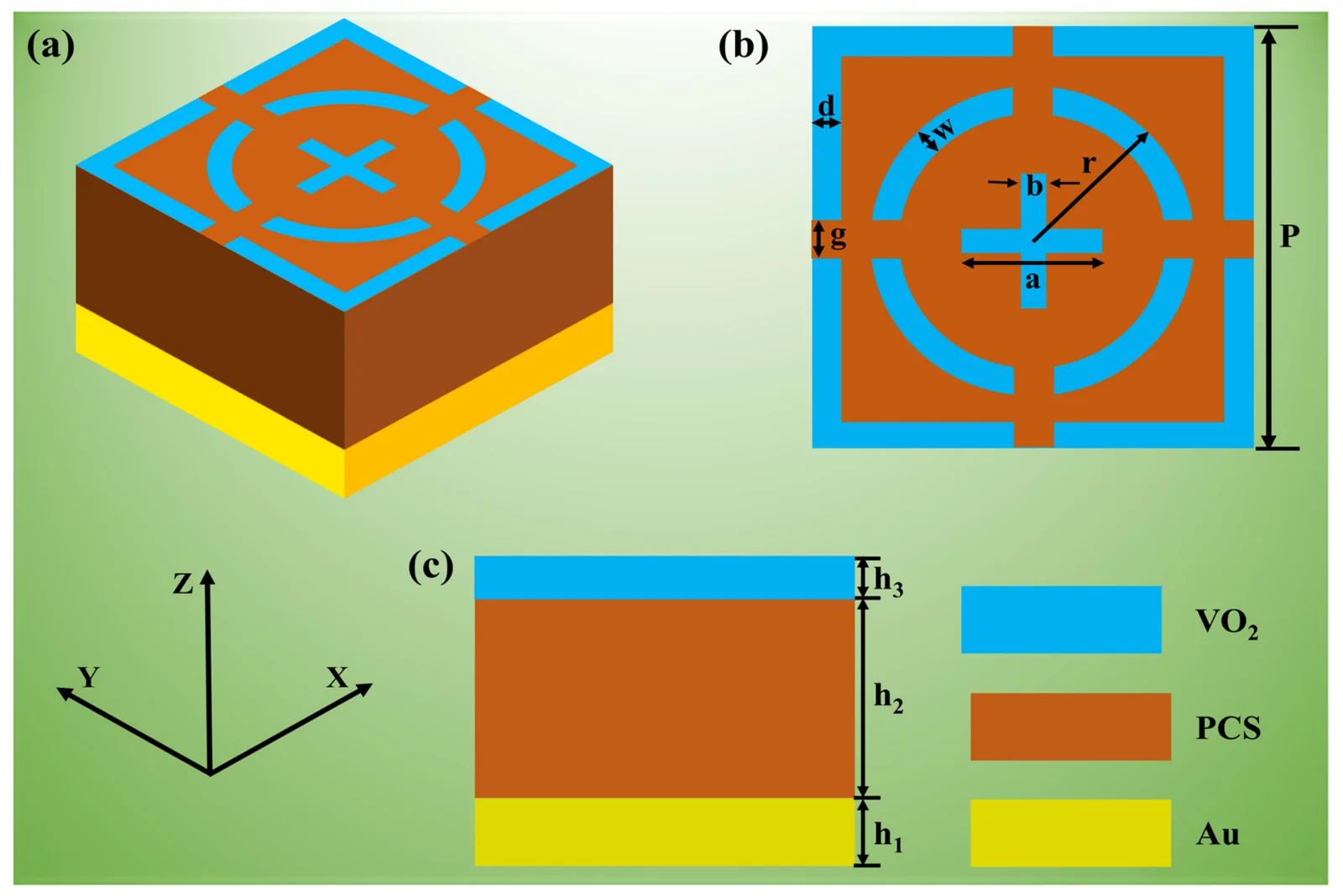
Geometry of the proposed VO_2_-based terahertz metasurface absorber: (**a**) three-dimensional view of the unit cell, (**b**) top view of the patterned VO_2_ resonator, and (**c**) cross-sectional view of the three-layer configuration.

**Figure 2:**
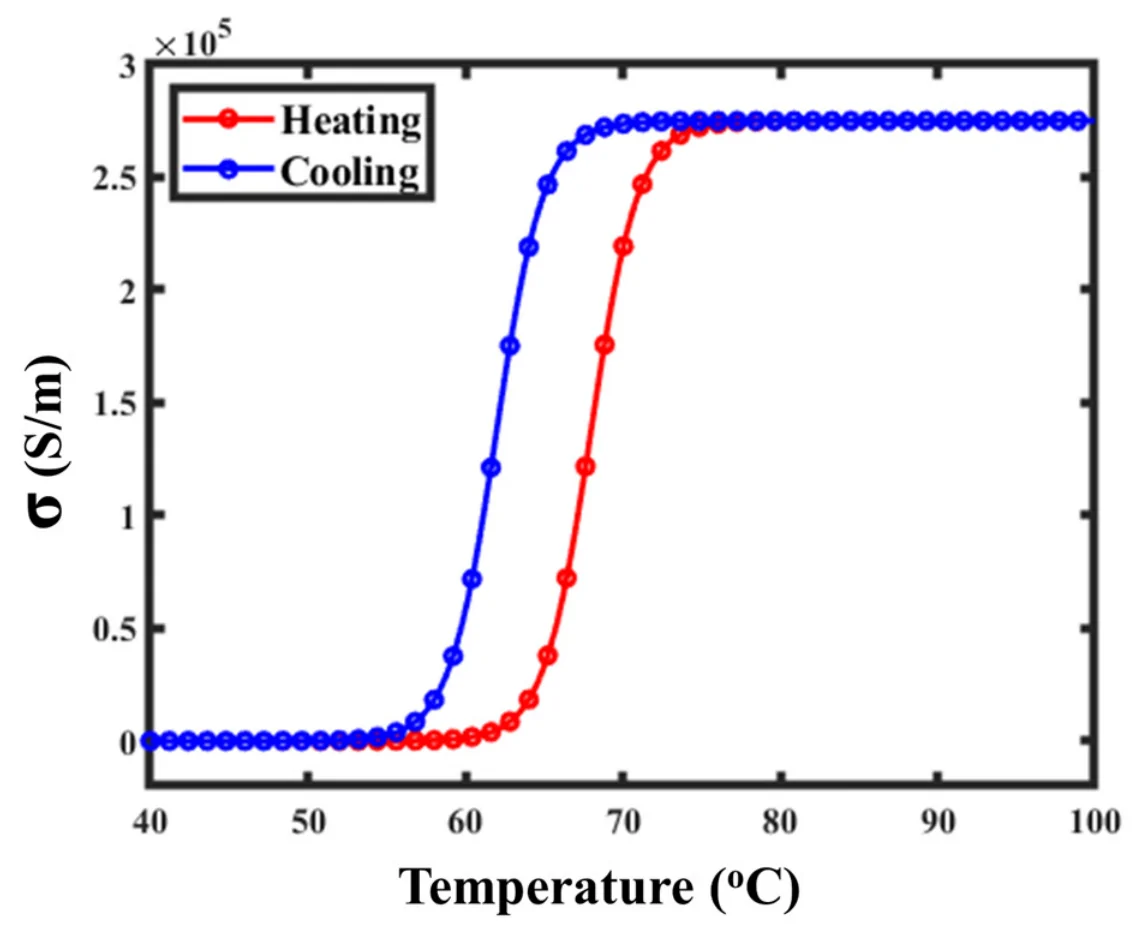
Temperature-dependent electrical conductivity of VO_2_ illustrating the hysteretic insulator-to-metal phase transition.

The geometry of the proposed terahertz metamaterial absorber is illustrated in Figure 1. The proposed absorber adopts a three-layer configuration consisting of a patterned VO_2_ resonator, a photonic crystal slab (PCS) dielectric spacer, and a continuous gold (Au) ground plane arranged along the propagation (z)-direction. As shown in Figure 1b, the metasurface consists of periodically repeated square unit cells with a periodicity of P along both the x- and y-directions. The patterned VO_2_ resonator comprises an outer square frame, a concentric circular ring, and a centrally positioned cross element arranged in a highly symmetric configuration. The outer square frame has a strip width of d and is separated from the inner resonator by a uniform gap of g. The circular ring is characterized by a radius of r and a strip width of w, whereas the central cross possesses an arm length of a and a strip width of b. To preserve structural symmetry, identical gaps of width g are introduced into both the square frame and the circular ring along the horizontal and vertical directions. The patterned VO_2_ resonator is separated from the metallic ground plane by a low-permittivity PCS dielectric spacer with a relative permittivity of ε_r_ = 1.56 and a thickness of h_2_ = 8.6 μm. The bottom layer consists of a continuous Au ground plane with a thickness of h_1_ = 0.2 μm and an electrical conductivity of 4.56 × 10^7^ S/m, while the thickness of the patterned VO_2_ layer is fixed at h_3_ = 0.4 μm. The relative permittivity of the PCS spacer (ε_r_ = 1.56) was adopted from prior photonic-crystal-slab-based terahertz absorber designs reported in the literature [48], where a low-index PCS layer is used to reduce the impedance mismatch with free space. The permittivity of VO_2_ is described by the Drude model given in Equations (1) and (2), with the insulating and metallic conductivities corresponding to the steady-state branches of the temperature-dependent hysteresis curve shown in Figure 2.

The optimized geometrical parameters of the proposed unit cell are summarized in Table 1.

Vanadium dioxide (VO_2_) is employed as the active resonator material owing to its reversible insulator-to-metal phase transition, which enables dynamic modulation of its electrical conductivity and, consequently, the electromagnetic response of the proposed absorber [41,42]. In the terahertz frequency regime, the electromagnetic response of VO_2_ is described using the Drude model [13,49], in which the complex permittivity is expressed as:

(1) $$\varepsilon \left(\omega \right)=\varepsilon_{\infty }-\frac{\omega_{p}^{2}(\sigma )}{\omega^{2}+\mathrm{i\gamma \omega}}$$

where *ε*_∞_ = 12 is the high-frequency dielectric constant, *γ* = 5.75 × 10^13^ rad/s is the carrier collision frequency, and *ω* denotes the angular frequency of the incident electromagnetic wave. The conductivity-dependent plasma frequency is given by:

(2) $$\omega_{p}^{2}\left(\sigma \right)=\frac{\sigma }{\sigma_{0}}\omega_{p}^{2}(\sigma_{0})$$

where σ_0_ = 3 × 10^5^ S/m is the reference conductivity, and *ω*_p_(σ_0_) = 1.4 × 10^15^ rad/s is the corresponding reference plasma frequency [41,50]. In the present study, the electrical conductivity (*σ*) is varied to represent different phases of VO_2_ during the thermally induced insulator-to-metal transition.

Figure 2 illustrates the temperature-dependent electrical conductivity of VO_2_ during the heating and cooling processes. At low temperatures, VO_2_ remains in the insulating phase with relatively low electrical conductivity. As the temperature approaches the phase-transition temperature of approximately 68 °C, the conductivity increases abruptly by several orders of magnitude, indicating the transition to the metallic phase. During cooling, the reverse transition occurs at a lower temperature, resulting in the characteristic thermal hysteresis behavior. This hysteresis was modeled using a Boltzmann-type sigmoidal fit [51]:

(3) $$\sigma \left(T\right)=\sigma_{\mathrm{ins}}+\frac{\sigma_{\mathrm{met}}-\sigma_{\mathrm{ins}}}{\left\{1+\exp[-(T-T_{c})/∆T]\right\}}$$

Distinct transition temperatures *T_c_* are used for the heating and cooling branches to capture the thermal hysteresis, consistent with the reported switching behavior of VO_2_. In the electromagnetic simulations, representative conductivity values extracted from the steady-state branches of the hysteresis curve were employed to model the insulating and metallic phases of VO_2_.

The absorptance *A*(*ω*) of the metamaterial absorber is calculated using the following relation [5,52]:

*A*(*ω*) = 1 − R(ω) − T(ω) (4)

where R(*ω*) and T(*ω*) denote the reflectance and transmittance, respectively, obtained directly from the simulated scattering parameters. Because the proposed absorber incorporates a continuous Au ground plane, electromagnetic transmission is effectively suppressed throughout the investigated terahertz frequency range.

The skin depth of the Au ground plane is given by:

(5) $$\delta =\sqrt{\frac{2}{\omega \mu_{0}\sigma {\mathrm{\varepsilon A}}_{u}}},\omega =2\mathrm{\pi f}$$

where μ_0_ is the permeability of free space, σ_Au_ is the electrical conductivity of gold, and *ω* is the angular frequency. Using σ_Au_ = 4.56 × 10^7^ S/m, the calculated skin depth ranges from approximately 43.4 nm at 2.95 THz to 23.2 nm at 10.36 THz, which is considerably smaller than the adopted Au thickness of 0.2 μm. Therefore, electromagnetic transmission through the ground plane is negligible (T ≈ 0), and the absorptance can be simplified as:

(6) $$A\left(\omega \right)\approx 1-{\left|S_{11}\right|}^{2},$$

where *S*_11_ represents the reflection coefficient of the absorber.

The electromagnetic response of the proposed absorber was simulated using the frequency-domain solver in CST Microwave Studio. Periodic boundary conditions were applied along the x- and y-directions to model an infinitely periodic metasurface, while open (add space) boundary conditions were assigned along the propagation (z)-direction to emulate free-space excitation. A normally incident plane wave propagating along the positive z-direction was employed as the excitation source. The polarization response was evaluated by rotating the incident electric-field vector, whereas the angular performance was investigated under transverse-electric (TE) and transverse-magnetic (TM) polarizations. A tetrahedral mesh was adopted throughout the simulations to ensure an appropriate balance between computational efficiency and numerical accuracy.

To quantitatively evaluate the broadband absorption performance, the relative bandwidth *(RBW)* is defined as:

(7) $$\mathrm{RBW}=2\times \frac{\left(f_{h}-f_{l}\right)}{\left(f_{h}+f_{l}\right)}\times 100\%,$$

where *f_h_* and *f_l_* correspond to the highest and lowest frequency limits over which the absorptance exceeds 90%. For the ultra-broadband region spanning 2.95–10.36 THz, the calculated *RBW* is approximately 111.35%. This wide bandwidth is associated with the combined contributions of multiple resonant responses, which support broadband impedance matching over the considered frequency range.

## 2. Results and Discussion

**Figure 3:**
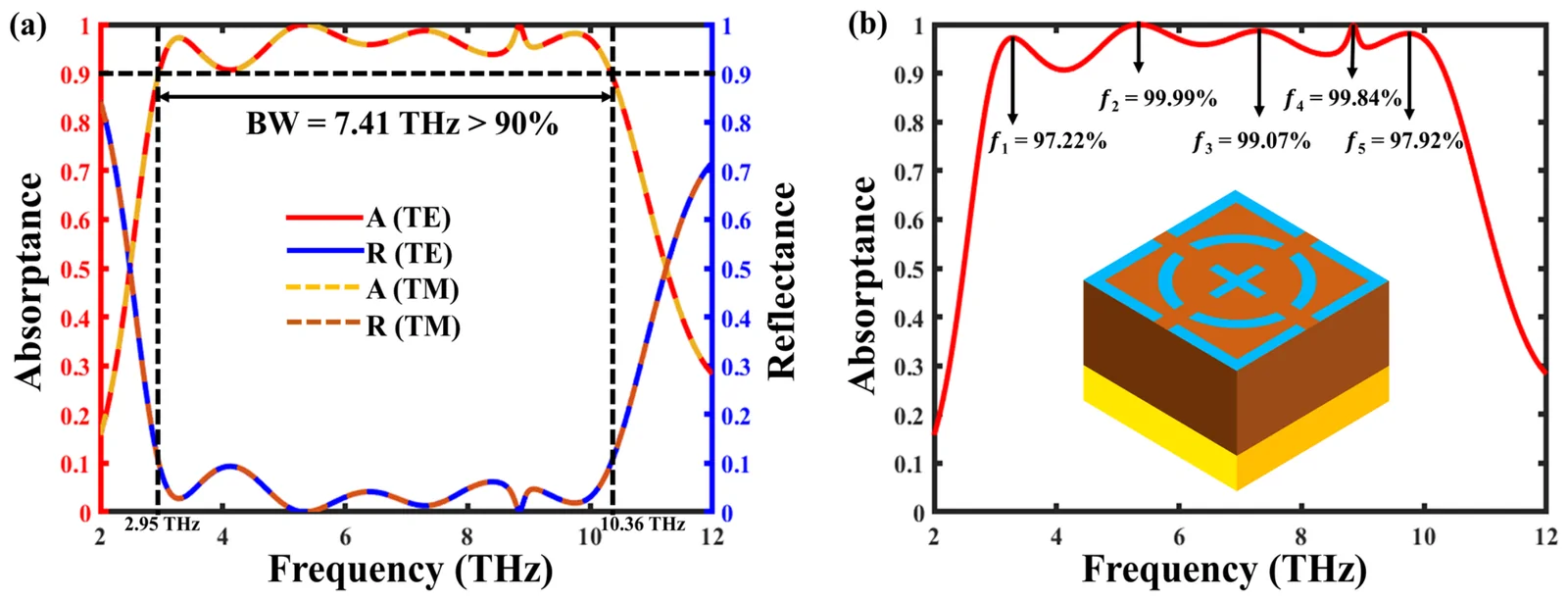
(**a**) Simulated absorptance and reflectance spectra of the proposed absorber under normal incidence with TE polarization. (**b**) Enlarged view of the five near-perfect absorption resonances located at *f*_1_ = 3.23 THz, *f*_2_ = 5.27 THz, *f*_3_ = 7.34 THz, *f*_4_ = 8.82 THz, *f*_5_ = 9.73 THz.

The absorption performance of the proposed VO_2_-based metamaterial absorber under normal-incidence TE-polarized excitation is presented in Figure 3. As shown in Figure 3a, the absorber exhibits an ultra-broadband response with five pronounced resonances at 3.23, 5.27, 7.34, 8.82, and 9.73 THz, achieving peak absorptance values of 97.22%, 99.99%, 99.07%, 99.84%, and 97.92%, respectively, as highlighted in Figure 3b. These resonances originate from the excitation and hybridization of multiple modes supported by the concentric VO_2_ resonator. Their spectral overlap produces a continuous absorption band with absorptance exceeding 90% over the frequency range of 2.95–10.36 THz, corresponding to a bandwidth of 7.41 THz. Although absorptance remains continuously above 90% across this range, the response is not perfectly flat: the deepest local minimum occurs at 4.12 THz, between the first and second resonances, where the absorptance falls to 90.64%, giving a peak-to-valley ripple of ΔA ≈ 9.35 percentage points relative to the highest peak (99.99% at 5.34 THz). Shallower dips of similar origin also occur near 6.39, 8.40 and 9.08 THz. This ripple reflects the discrete spacing of the five hybridized resonant modes, which remain individually resolvable rather than merging into a single, fully flat-top response. Moreover, the reflectance remains close to zero throughout this range, indicating efficient coupling of incident electromagnetic energy into the absorber, where it is dissipated within the lossy VO_2_ resonator, resulting in near-unity absorption.

To further elucidate the absorption mechanism, the impedance characteristics of the proposed absorber were analyzed. For metallic-backed metamaterial absorbers, near-perfect absorption is achieved when the effective input impedance is matched to that of free space, thereby minimizing reflection at the air–absorber interface. The normalized effective impedance is retrieved from the simulated scattering parameters as:

(8) $$Z_{r}\left(\omega \right)=\sqrt{\frac{\left[1+{S_{11}}^{2}\right]-S_{21}^{2}}{\left[1-{S_{11}}^{2}\right]-S_{21}^{2}}.}$$

**Figure 4:**
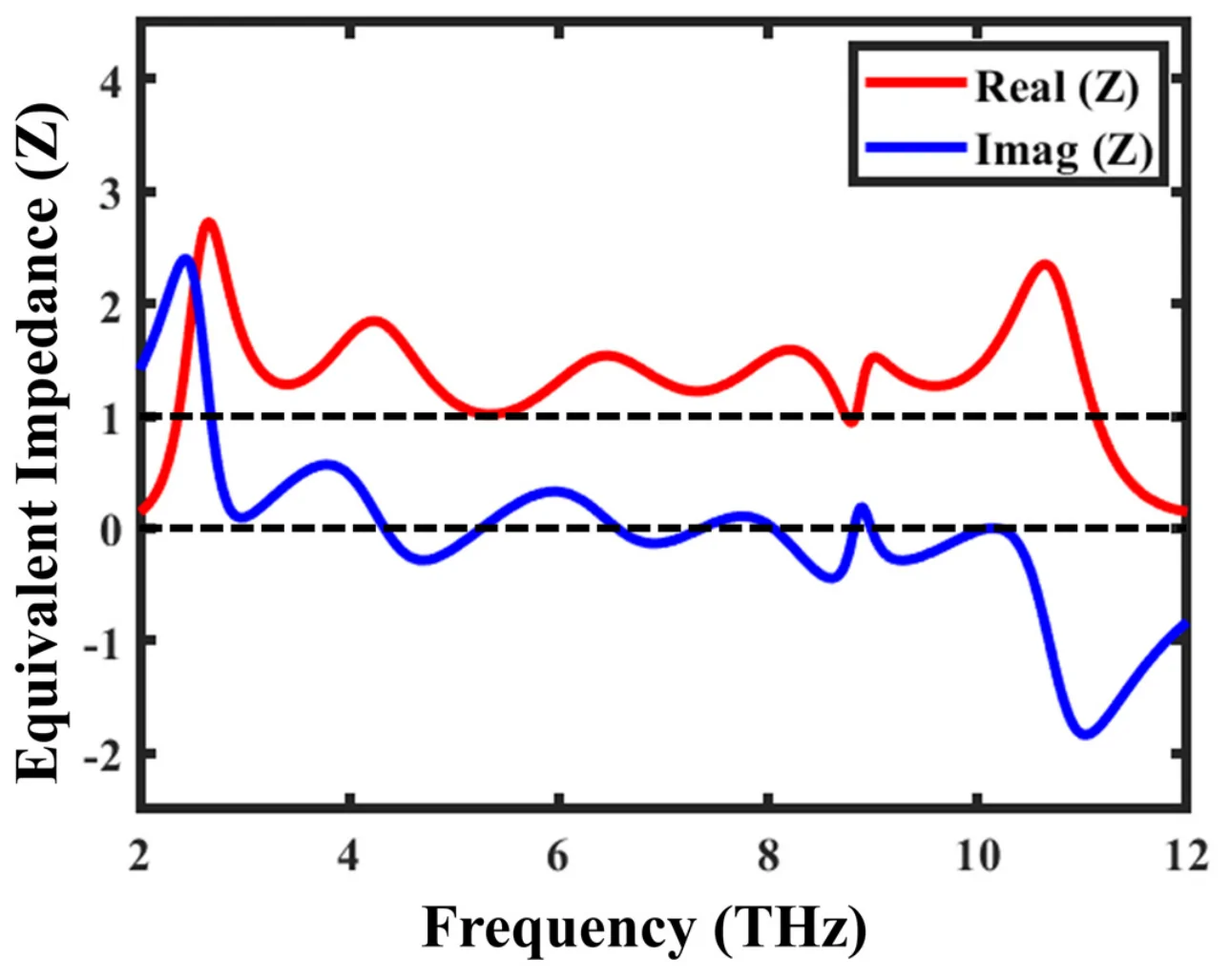
Normalized real and imaginary parts of the effective impedance under TE-polarized excitation, demonstrating near-ideal impedance matching at the five absorption resonance frequencies.

The reflection coefficient *S*_11_ and transmission coefficient *S*_21_ were obtained directly from the frequency-domain CST simulation. The normalized effective impedance was subsequently retrieved from these simulated scattering parameters using Equation (6). Since the continuous Au ground plane effectively suppresses transmission, *S*_21_ is approximately zero over the investigated frequency range, and the impedance response is therefore primarily determined by the simulated reflection coefficient *S*_11_. As shown in Figure 4, the real part of the normalized impedance remains close to unity, while the imaginary part approaches zero throughout the broadband operating range (2.95–10.36 THz), particularly at the resonance frequencies. These results confirm effective impedance matching with free space, which suppresses reflection and enables the observed ultra-broadband absorption.

**Figure 5:**
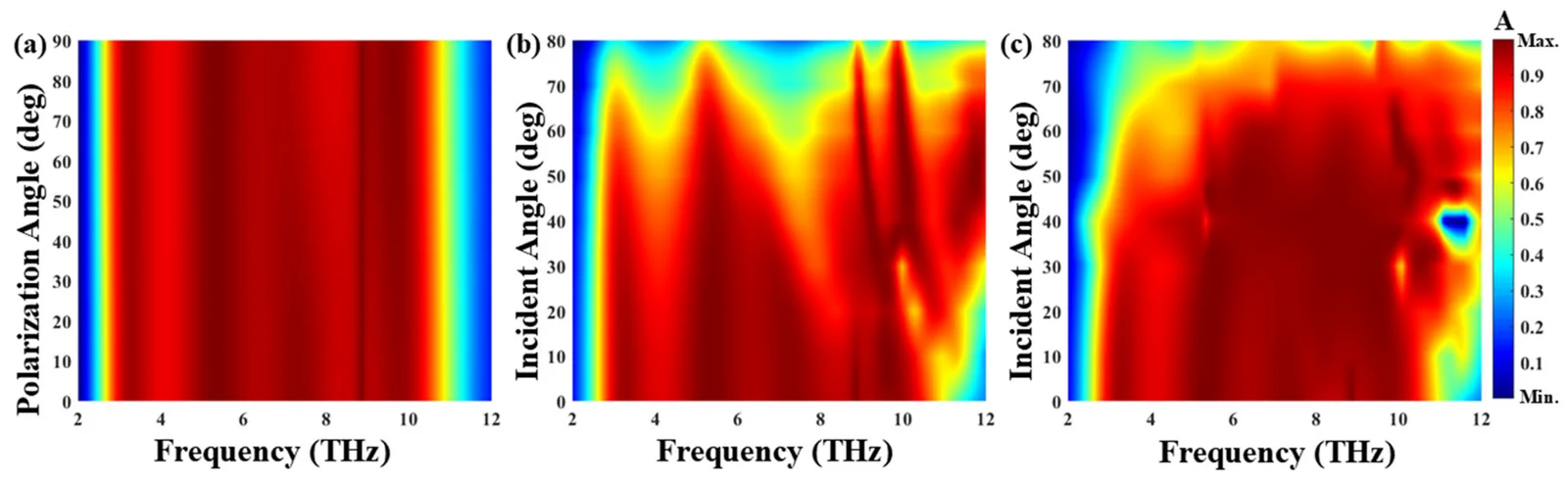
Absorptance spectra of the proposed absorber under (**a**) varying polarization angles at normal incidence, confirming polarization insensitivity; (**b**) oblique TE incidence and (**c**) oblique TM incidence demonstrating robust angular stability. In each panel, color denotes the absorptance magnitude (A) according to the color bar.

The polarization and angular responses of the proposed absorber are shown in Figure 5, where the color scale in each panel represents the magnitude of the simulated absorptance (A), ranging from minimum (dark blue) to maximum (dark red), as indicated by the color bar. As illustrated in Figure 5a, the absorptance spectra remain nearly unchanged as the polarization angle varies from 0° to 90°, with negligible resonance shifts and absorptance exceeding 90% across the operating bandwidth. This polarization-insensitive behavior arises from the highly symmetric geometry of the concentric VO_2_ resonator, which produces nearly identical electromagnetic responses for orthogonal polarization states. The angular performance of the absorber under TE and TM polarized oblique incidence is presented in Figure 5b and Figure 5c, respectively. As the incidence angle increases from 0° to 60°, the broadband absorption response is largely preserved, with only minor degradation observed at larger angles. Even at 50°, absorptance remains above 90% over most of the operating range, demonstrating excellent angular robustness.

**Figure 6:**
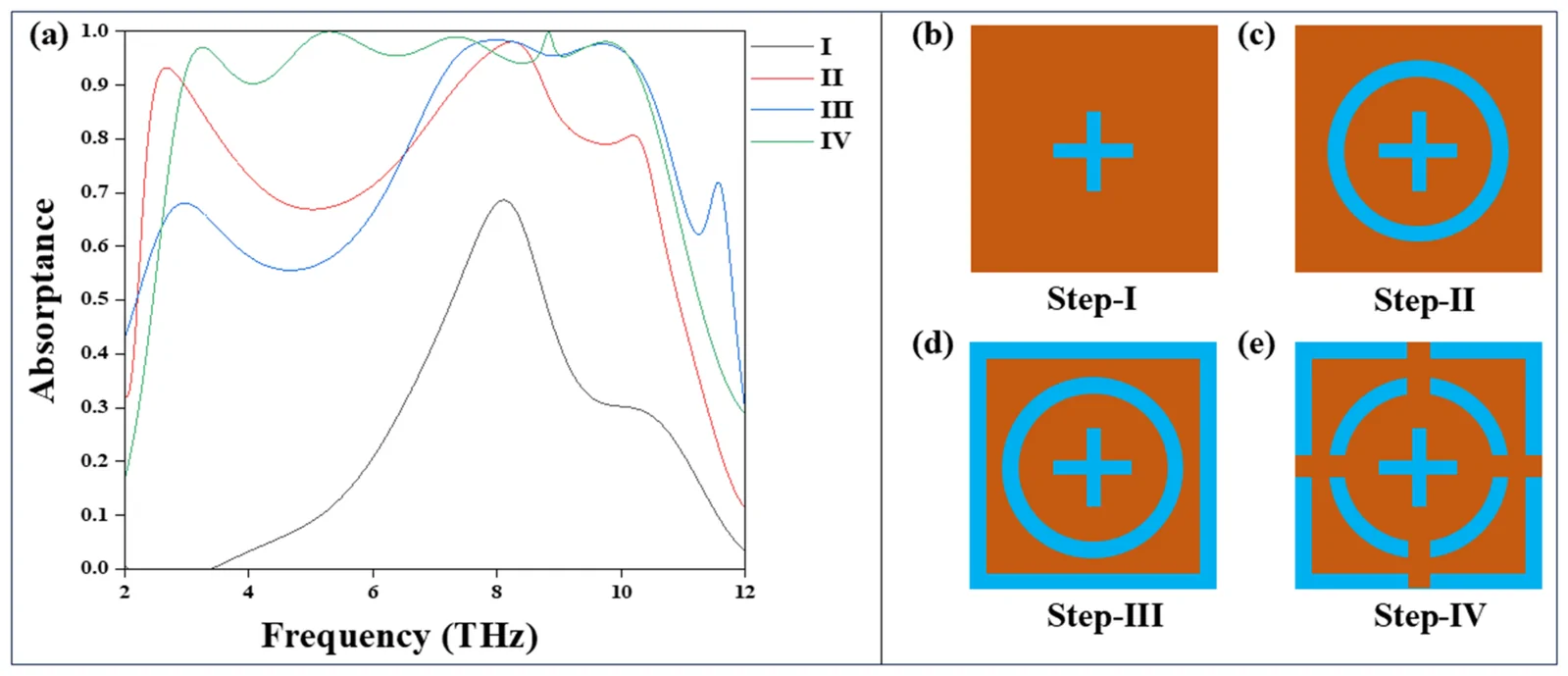
(**a**) Simulated absorptance spectra of the four VO_2_ resonator configurations. (**b**–**e**) Evolution of the resonator geometry from Step-I (cross resonator), Step-II (cross with concentric ring), Step-III (square frame with concentric ring and cross), and Step-IV (final optimized resonator with symmetric gaps).

To elucidate the origin of the broadband absorption response, four VO_2_ resonator configurations were systematically examined, as shown in Figure 6a, with their corresponding geometries presented in Figure 6b–e. Case-I consists of a central cross resonator, Case-II incorporates a concentric circular ring, Case-III introduces an outer square frame, and Case-IV represents the final optimized design with symmetric gaps in both the ring and frame. The Case-I structure exhibits a relatively narrow absorption band owing to the limited number of supported resonant current paths. The addition of the concentric ring in Case-II introduces extra resonant modes and broadens the absorption response. In Case-III, the outer square frame enhances near-field coupling and promotes stronger mode hybridization, resulting in further bandwidth expansion. Finally, the optimized gapped configuration (Case-IV) improves the coupling among resonant elements and facilitates effective impedance matching, enabling multiple resonances to merge into a continuous broadband response. Consequently, the final design achieves the broadest absorption bandwidth and the most uniform spectral profile. These results demonstrate that the combined effects of the concentric ring, square frame, and optimized gaps are critical for realizing the observed ultra-broadband absorption.

It should be emphasized that the central design principle of this work is not the use of a cross, ring, and square-frame resonator per se, but the deliberate use of coupling gaps as a design variable to control the interaction between these resonators. As shown by the progression from Case I to Case IV, the same three geometric elements do not, by themselves, produce the reported bandwidth; it is the symmetric gaps introduced into the ring and frame in Case IV that govern the strength of near-field coupling between the resonators and drive their individual modes to hybridize into a single, continuous, impedance-matched absorption band. This gap-controlled coupling strategy, rather than the constituent shapes, is what distinguishes the present design from prior VO_2_-based absorbers that combine similar geometric elements. As summarized in Table 2, this strategy enables the highest relative bandwidth (111.35%) among the compared single-active-layer VO_2_ absorbers, without additional structural or material complexity, while simultaneously preserving polarization insensitivity and wide angular stability (up to 50° for both TE and TM polarization).

**Figure 7:**
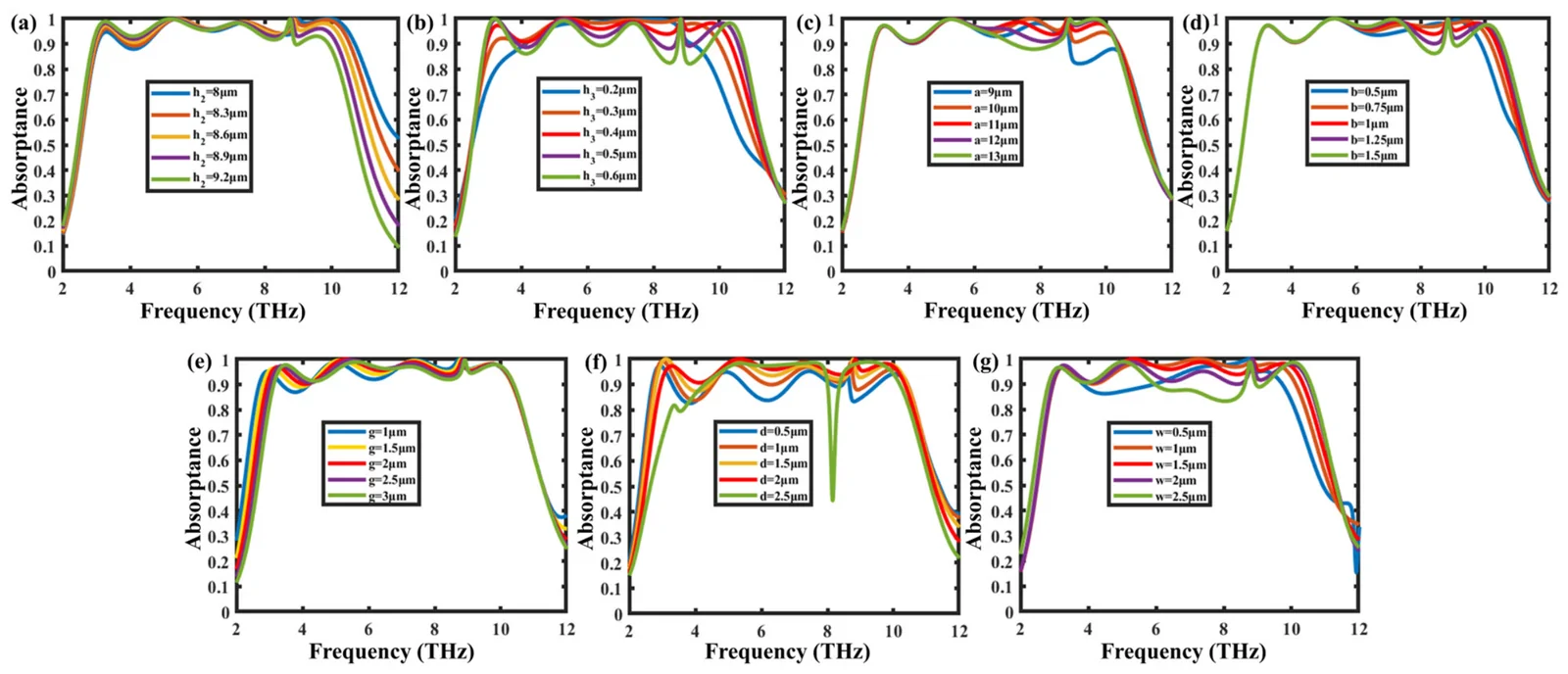
Parametric absorptance analysis of key design parameters: (**a**) PCS spacer thickness h_2_, (**b**) VO_2_ thickness h_3_, (**c**) central cross length a, (**d**) central cross width b, (**e**) gap width g, (**f**) square-frame width d, (**g**) ring width w.

Figure 7 presents the parametric analysis of the key geometrical parameters to evaluate their influence on the absorption response and to identify the optimized design. As shown in Figure 7a, varying the thickness of the photonic crystal slab (PCS) spacer (h_2_) modifies the phase delay and magnetic-field confinement between the VO_2_ resonator and the metallic ground plane, resulting in slight spectral shifts while preserving broadband operation. Figure 7b illustrates the effect of the VO_2_ thickness (h_3_), where increasing the resonator thickness enhances electromagnetic loss and field confinement, thereby improving absorption uniformity. The effects of the central cross dimensions are shown in Figure 7c,d, corresponding to the cross-arm length (a) and width (b), respectively. Variations in these parameters alter the resonant current paths and consequently influence the resonance frequencies and spectral flatness. As shown in Figure 7e, the gap width (g) strongly affects the near-field interaction between adjacent resonant elements. A smaller gap enhances electromagnetic coupling and mode hybridization, promoting greater spectral overlap, whereas a larger gap weakens the interaction and reduces the broadband response. The influence of the square-frame width (d) and ring width (w) is presented in Figure 7f and Figure 7g, respectively. Variations in these parameters modify the surface-current distribution and resonant behavior, leading to changes in impedance matching and absorption bandwidth. Overall, the absorber maintains excellent broadband performance over a wide range of parameter variations, indicating reasonable tolerance to fabrication deviations. The optimized dimensions are h_2_ = 8.6 μm, h_3_ = 0.4 μm, a = 11 μm, b = 1 μm, d = 1 μm, g = 2 μm, r = 12 μm, and w = 1.53 μm.

The polarization insensitivity of the absorber at normal incidence originates from the four-fold rotational symmetry of the unit cell. Fabrication errors that preserve this symmetry, such as uniform over- or under-etching or thickness variations, cannot introduce polarization dependence and only shift the absorption band, as shown by the parametric study in Figure 7. Errors that affect only one axis would break the symmetry and introduce a small difference between x- and y-polarized waves. Because each polarization is mainly sensitive to the dimensions along its own axis, this difference is expected to be bounded by the spectral changes in Figure 7 for the same dimensional change. The parameter variations in Figure 7 (0.25–1 μm) are comparable to or larger than the dimensional accuracy of conventional photolithography and electron-beam lithography (a few tens of nanometres). The overlap of five resonances also provides a margin, since a shift of one resonance is partly compensated by its neighbours. The smallest features (d, b and g) are the most sensitive, and higher-resolution lithography is recommended for them. A full simulation of symmetry-breaking fabrication errors, together with experimental verification, is left for future work.

**Figure 8:**
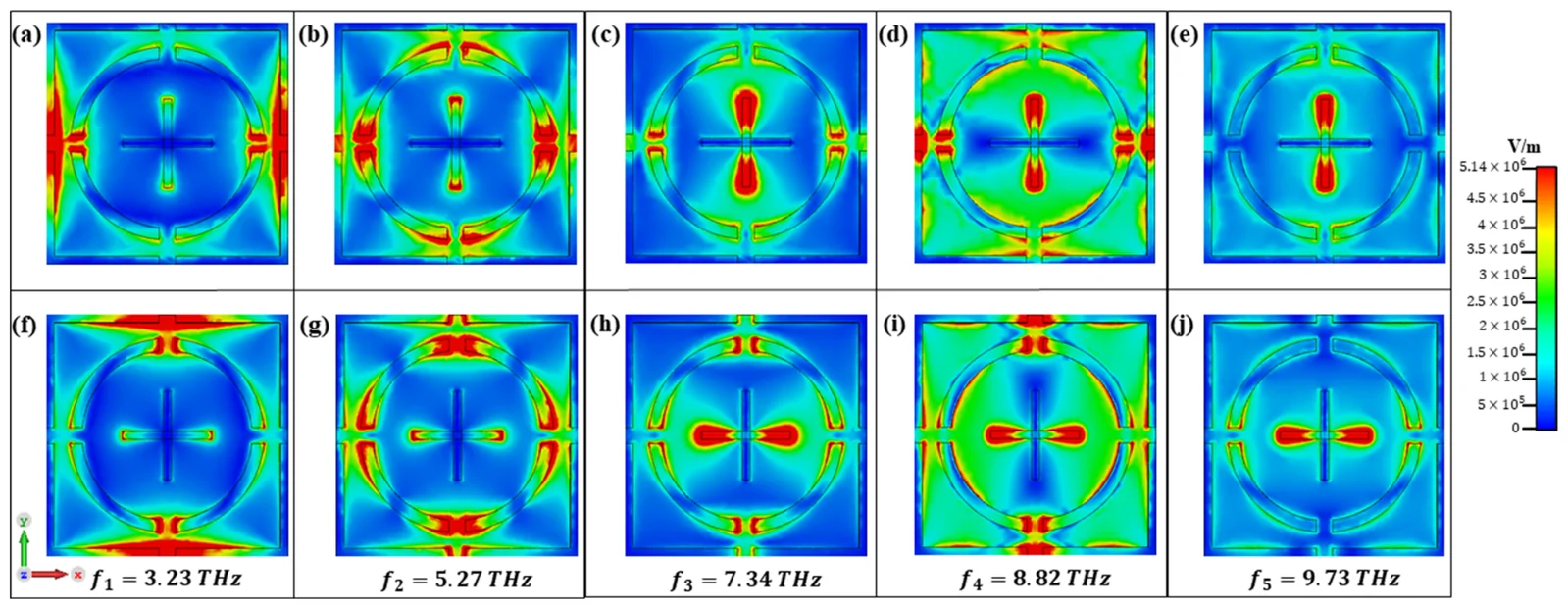
Simulated near-field distributions of the proposed absorber under TE polarization: (**a**–**e**) E-field magnitude at *f*_1_ = 3.23 THz, *f*_2_ = 5.27 THz, *f*_3_ = 7.34 THz, *f*_4_ = 8.82 THz, *f*_5_ = 9.73 THz; (**f**–**j**) corresponding E-field distributions at the same frequencies under TM-polarized excitation.

To further clarify the absorption mechanism, the electric- and magnetic-field distributions of the proposed VO_2_-based metamaterial absorber were analyzed under TE-polarized excitation at the five resonance frequencies, as shown in Figure 8. Figure 8a–e present the electric-field (|E|) distributions at *f*_1_ = 3.23 THz, *f*_2_ = 5.27 THz, *f*_3_ = 7.34 THz, *f*_4_ = 8.82 THz and *f*_5_ = 9.73 THz, respectively. Strong electric-field localization is observed along the edges of the concentric VO_2_ resonator, indicating significant charge accumulation and electric-dipole excitation. As the frequency increases, the dominant field concentration progressively shifts from the outer square frame to the coupling region between the square frame and circular ring, then to the ring–cross interface, and finally to the central cross resonator, revealing that different resonator elements contribute to distinct resonant modes whose collective interaction gives rise to the broadband absorption response. Figure 8f–j show the corresponding E-field distributions under TM-polarized excitation are at the same frequencies. The field patterns are, in each case, a 90-rotated counterpart of the TE-polarization distributions in Figure 8a–e, with comparable peak field magnitudes. This near-identical, symmetry-related field response under both polarizations provides direct near-field confirmation of the polarization-insensitive behavior demonstrated in the far-field absorptance spectra in Figure 5a, and is consistent with the fourfold rotational symmetry of the resonator geometry. Overall, the field distributions confirm that the observed ultra-broadband response originates from the hybridization of multiple resonant modes supported by the concentric VO_2_ resonator.

This assignment is corroborated by the parametric analysis in Figure 7: the frame width d and gap width g most strongly shift the lower resonances (f_1_, f_2_), the ring width w most strongly affects f_2_ and f_4_, and the cross dimensions a and b most strongly affect the upper resonances (f_4_, f_5_). Notably, although f_3_ is spatially localized on the cross in Figure 8, its frequency is governed mainly by the ring and frame parameters (w, d, g) rather than by the cross dimensions, indicating that its position results from loading by the surrounding coupled elements rather than from the cross acting as an isolated resonator. Because the frame, ring, and cross are concentric and separated only by the gap g, their near-fields overlap, as seen directly in the simultaneous ring–cross activation at f_4_; this coupling shifts and interleaves the five resonances into a closely spaced sequence, allowing their individual absorption bands to merge into the continuous response in Figure 3. This gap, the key variable between Case I and Case IV in Figure 6, controls the coupling strength and therefore how fully the five resonances merge.

**Figure 9:**
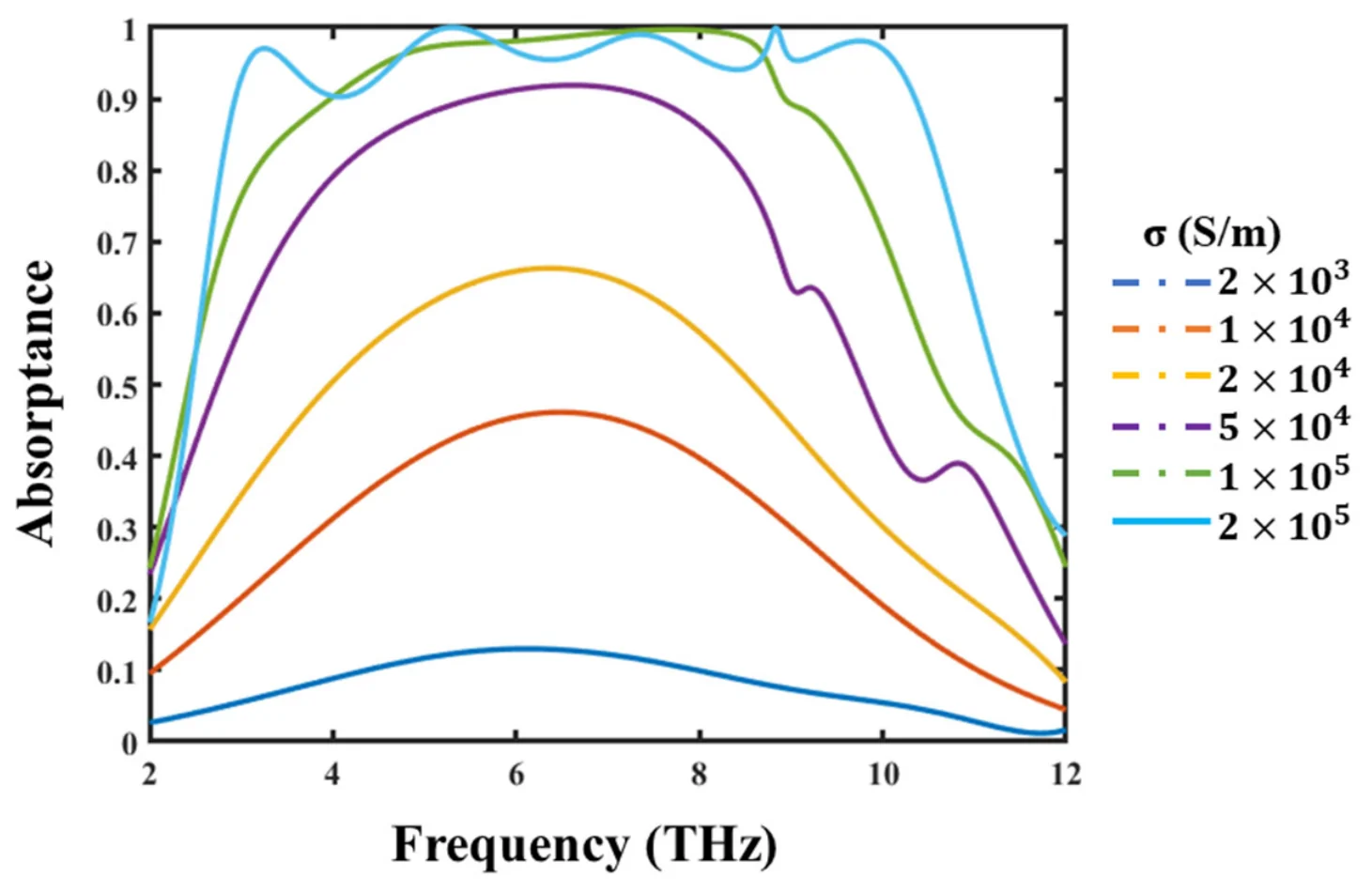
Absorptance spectra of the VO_2_-based terahertz absorber for different VO_2_ conductivities (2 × 10^2^ to 2 × 10^5^ S/m), illustrating tunable broadband absorption enabled by the insulator-to-metal phase transition of VO_2_.

Figure 9 shows the absorptance spectra of the proposed absorber as the electrical conductivity of VO_2_ varies from 2 × 10^2^ to 2 × 10^5^ S/m, corresponding to its thermally induced insulator-to-metal phase transition. At low conductivity, VO_2_ exhibits insulating behavior, resulting in weak interaction with the incident electromagnetic waves and limited energy dissipation, which leads to relatively low absorptance. As the conductivity increases, the resonant response of the patterned VO_2_ resonator is progressively strengthened, giving rise to enhanced electromagnetic confinement and a broader absorption bandwidth. When the conductivity reaches 2 × 10^5^ S/m, corresponding to the metallic phase, the absorber exhibits broadband absorptance exceeding 90% over a wide terahertz frequency range. This behavior is attributed to the strong surface-current excitation supported by the highly conductive VO_2_ resonator and the improved impedance matching with free space, which effectively suppresses reflection and enhances energy dissipation. These results demonstrate that the absorption response can be continuously tuned through the phase-transition characteristics of VO_2_, highlighting the potential of the proposed absorber for thermally reconfigurable terahertz applications.

The practical thermal tuning of the proposed absorber can be realized through the temperature-dependent phase transition of VO_2_. As shown in Figure 2, increasing temperature drives VO_2_ from its insulating state toward the metallic state, accompanied by a significant increase in electrical conductivity and a characteristic thermal hysteresis during heating and cooling. The resulting change in the electromagnetic properties of VO_2_ modifies its interaction with the incident THz wave, leading to the absorption variation shown in Figure 9. It should be noted that the intermediate conductivity values in Figure 9 represent different effective states during the phase transition and should not be interpreted as independently controlled homogeneous conductivity values. In practical implementation, controlled heating or cooling can be used to regulate the VO_2_ phase transition, while the hysteresis behavior should be considered when controlling intermediate states reproducibly. Thus, the conductivity-dependent simulations illustrate the evolution of the absorption response associated with the thermally induced VO_2_ phase transition.

From a fabrication perspective, the proposed VO_2_ absorber is compatible with standard microfabrication techniques commonly used for terahertz devices. The minimum feature sizes, including the square-frame width (d = 1 μm), ring width (w = 1.53 μm), and gap width (g = 2 μm), are within the capabilities of conventional photolithography, while electron-beam lithography may be employed when higher precision is required. A feasible fabrication process involves deposition of the Au ground plane, formation of the PCS dielectric spacer, deposition of the VO_2_ thin film, and subsequent patterning of the VO_2_ resonator through lithography and etching. These well-established techniques indicate the practical feasibility of experimentally realizing the proposed absorber. Furthermore, the parametric analysis in Figure 7 shows that the broadband response is tolerant to dimensional variations comparable to the resolution of these techniques.

To further evaluate its performance, Table 2 compares the proposed absorber with recently reported VO_2_-based terahertz metamaterial absorbers.

The proposed design achieves an absorption bandwidth of 7.41 THz (2.95–10.36 THz) with absorptance exceeding 90%, corresponding to a relative bandwidth of 111.35%, which is among the highest reported for VO_2_-based absorbers. In addition to its broadband response, the absorber exhibits polarization-insensitive behavior and maintains stable absorption up to an incident angle of 50° under both TE and TM polarizations. The simple three-layer VO_2_–PCS–Au configuration supports multiple resonant modes that collectively contribute to the enhanced absorption performance. Moreover, the phase-transition characteristics of VO_2_ enable dynamic tuning of the absorption response through conductivity modulation without modifying the device geometry. These features demonstrate that the proposed absorber combines broadband operation, structural simplicity, angular stability, polarization independence, and tunability, making it a promising candidate for practical terahertz applications.

The combination of properties demonstrated by the proposed absorber, namely ultra-broadband absorption (2.95–10.36 THz), polarization insensitivity, wide-angle stability, and thermally reversible tunability, makes it well suited to several terahertz application areas. In terahertz imaging and non-destructive sensing, broadband and polarization-independent absorption allows consistent response across the illumination spectrum and object orientation, without requiring a polarization-matched source. The reversible VO_2_ switching further enables the absorber to be reconfigured between a low-absorption (metallic, high-conductivity) and a broadband high-absorption (insulating, low-conductivity) state, which can be exploited for amplitude modulation in terahertz communication links or for tunable terahertz filters and switches. The broadband, angle-stable absorption is also relevant to electromagnetic-interference shielding and adaptive terahertz stealth coatings, where consistent absorption over a wide range of viewing angles is required. Finally, because the absorption resonances are sensitive to the local dielectric environment near the resonator, the structure may also serve as a platform for terahertz biosensing or material-characterization applications, where a shift in the resonance frequencies can be used to detect changes in an analyte placed near the metasurface. We note that these are proposed application directions based on the simulated performance, and experimental demonstration in each of these contexts is left for future work.

## 3. Conclusions

In this work, a compact VO_2_-based terahertz metamaterial absorber was numerically designed and investigated. The proposed structure, consisting of a patterned VO_2_ resonator, a PCS dielectric spacer, and a metallic ground plane, achieves ultra-broadband absorption with absorptance exceeding 90% over a wide terahertz frequency range. The excellent absorption performance originates from the synergistic effects of multiple resonant modes, strong electromagnetic field confinement, and effective impedance matching with free space. The absorption mechanism was verified through impedance analysis and near-field distributions, which revealed efficient electromagnetic energy localization and dissipation within the resonator structure. Furthermore, the absorber exhibits good tolerance to geometrical variations, indicating its feasibility for practical fabrication. Owing to its four-fold symmetric configuration, the ideal structure maintains polarization-insensitive operation under normal incidence and stable absorption performance over a broad range of incident angles. In addition, the reversible insulator-to-metal phase transition of VO_2_ enables dynamic tuning of the absorption response through conductivity modulation, providing active control without altering the device geometry. The combination of ultra-broadband absorption, angular stability, polarization independence, structural simplicity, and thermal tunability makes the proposed absorber a promising candidate for the terahertz application areas discussed above.

## Figures and Tables

**Table 1:** Optimized geometrical parameters of the proposed VO_2_ absorber unit cell.

Parameter	Description	Value (µm)
P	Unit-cell periodicity	30
d	Square-frame width	1
g	Gap width	2
r	Ring radius	12
w	Ring width	1.53
a	Cross-arm length	11
b	Cross-arm width	1
h_1_	Au ground thickness	0.2
h_2_	PCS thickness	8.6
h_3_	VO_2_ thickness	0.4

**Table 2:** Performance comparison of the proposed VO_2_-based terahertz absorber with recently reported VO_2_-based terahertz metamaterial absorbers.

Ref.	Abs. Bandwidth (THz)	Absorption Rate	Relative Bandwidth (%)	Ripple ΔA (%)	Material Structure	Angular Stability	Polarization Insensitivity	Year
[41]	2.7	>90%	94.7	N/R	VO_2_-TOPAS-Au	60°	yes	2024
[53]	6.0	>90%	97.2	N/R	VO_2_-SiO_2_-Au	45°	yes	2024
[54]	5.19	>90%	99.8	N/R	VO_2_-PTFE-Au	45°	no	2025
[18]	6.35	>90%	106	N/R	VO_2_-Quartz-Au	45°	yes	2025
[55]	4.90	>90%	93.16	N/R	VO_2_-Quartz-Au	70°	yes	2023
[56]	5.10	>90%	79.20	N/R	VO_2_-TOPAS-Au	—	—	2023
[57]	5.10	>90%	86.5	N/R	VO_2_-Al_2_O_3_-Au	70°	yes	2023
[58]	5.5	>90%	75.9	N/R	VO_2_-MF_2_-Au	60°	yes	2023
[59]	3.30	>90%	84.6	N/R	VO_2_-MPA-Au	—	yes	2021
[60]	4.26	>90%	82.9	N/R	VO_2_-SiO_2_-Au	45°	no	2022
[61]	5.08	>90%	93.16	N/R	VO_2_-Quartz-Au	70°	yes	2023
[62]	4.70	>90%	87.19	N/R	VO_2_-Polyimide-Au	60°	yes	2025
[63]	6.67	>90%	106.6	N/R	VO_2_-PDMS-Au	60°	yes	2026
This Work	7.41	>90%	111.35	9.35	VO_2_-PCS-Au	50°	yes	2026

N/R: not reported in the cited work. Ripple ΔA (%) is the peak-to-valley absorptance variation within the ≥90% band.

## Data Availability

The authors confirm that the data supporting the findings of this study are available within the article.
